# Predicting preterm birth through vaginal microbiota, cervical length, and WBC using a machine learning model

**DOI:** 10.3389/fmicb.2022.912853

**Published:** 2022-08-02

**Authors:** Sunwha Park, Jeongsup Moon, Nayeon Kang, Young-Han Kim, Young-Ah You, Eunjin Kwon, AbuZar Ansari, Young Min Hur, Taesung Park, Young Ju Kim

**Affiliations:** ^1^Department of Obstetrics and Gynecology, College of Medicine, Ewha Medical Research Institute, Ewha Womans University, Seoul, South Korea; ^2^Interdisciplinary Program in Bioinformatics, Seoul National University, Seoul, South Korea; ^3^Department of Obstetrics and Gynecology, College of Medicine, Yonsei University, Seoul, South Korea; ^4^Department of Statistics, Seoul National University, Seoul, South Korea

**Keywords:** preterm birth, vaginal microbiome, pregnancy, 16s ribosomal RNA metagenome sequencing, cervicovaginal fluid, machine learning, microbial-marker

## Abstract

An association between the vaginal microbiome and preterm birth has been reported. However, in practice, it is difficult to predict premature birth using the microbiome because the vaginal microbial community varies highly among samples depending on the individual, and the prediction rate is very low. The purpose of this study was to select markers that improve predictive power through machine learning among various vaginal microbiota and develop a prediction algorithm with better predictive power that combines clinical information. As a multicenter case–control study with 150 Korean pregnant women with 54 preterm delivery group and 96 full-term delivery group, cervicovaginal fluid was collected from pregnant women during mid-pregnancy. Their demographic profiles (age, BMI, education level, and PTB history), white blood cell count, and cervical length were recorded, and the microbiome profiles of the cervicovaginal fluid were analyzed. The subjects were randomly divided into a training (*n* = 101) and a test set (*n* = 49) in a two-to-one ratio. When training ML models using selected markers, five-fold cross-validation was performed on the training set. A univariate analysis was performed to select markers using seven statistical tests, including the Wilcoxon rank-sum test. Using the selected markers, including *Lactobacillus* spp., *Gardnerella vaginalis*, *Ureaplasma parvum*, *Atopobium vaginae*, *Prevotella timonensis*, and *Peptoniphilus grossensis*, machine learning models (logistic regression, random forest, extreme gradient boosting, support vector machine, and GUIDE) were used to build prediction models. The test area under the curve of the logistic regression model was 0.72 when it was trained with the 17 selected markers. When analyzed by combining white blood cell count and cervical length with the seven vaginal microbiome markers, the random forest model showed the highest test area under the curve of 0.84. The GUIDE, the single tree model, provided a more reasonable biological interpretation, using the 10 selected markers (*A. vaginae*, *G. vaginalis*, *Lactobacillus crispatus*, *Lactobacillus fornicalis*, *Lactobacillus gasseri*, *Lactobacillus iners*, *Lactobacillus jensenii*, *Peptoniphilus grossensis*, *P. timonensis*, and *U. parvum*), and the covariates produced a tree with a test area under the curve of 0.77. It was confirmed that the association with preterm birth increased when *P. timonensis* and *U. parvum* increased (AUC = 0.77), which could also be explained by the fact that as the number of *Peptoniphilus lacrimalis* increased, the association with preterm birth was high (AUC = 0.77). Our study demonstrates that several candidate bacteria could be used as potential predictors for preterm birth, and that the predictive rate can be increased through a machine learning model employing a combination of cervical length and white blood cell count information.

## Introduction

Preterm birth (PTB) is defined as delivery at less than 37 weeks of gestation, and prematurity from PTB is a major cause of morbidity and mortality among infants (Goldenberg et al., 2008). The risk factors for PTB are influenced by ethnicity, low socioeconomic status, maternal weight, smoking, periodontal status, and underlying diseases (Koullali et al., 2016). Due to the increase in elderly pregnant women and pregnant women with various underlying diseases, PTB is increasing, and efforts are being made to predict and prevent it (Newnham et al., 2017; Ananth et al., 2018). Among PTB, spontaneous PTB accounts for 70–75% of all cases, and one-third of them are caused by intra-amniotic infection, an infection of the tissues surrounding the fetus (Goldenberg et al., 2008; Chan, 2014). Microorganisms that cause these intra-amniotic infections (*Ureaplasma* spp., *Gardnerella vaginalis*) show similar patterns to those of the lower genital tract, and are known to induce uterine contractions and premature rupture of membranes due to an inflammatory response caused by the ascending infection (Romero et al., 2014; Bennett et al., 2020; Park et al., 2020). Therefore, methods to evaluate the risk of PTB by microscopy, culture, and polymerase chain reaction (PCR) are being carried out in clinical practice. Furthermore, with the development of 16s rRNA metagenome sequencing, it has become possible to analyze not only pathogens but also the microbial community, that is, the microbiome (Yoo et al., 2016; Hur et al., 2021).

In pregnant women, an increase in *Lactobacillus* is known to be associated with term birth (TB), whereas an increase in *G. vaginalis*, *Ureaplasma* spp., *Prevotella* spp., *Atopobium vaginae, Peptoniphilus* spp., *Staphylococcus aureus, Streptococcus* spp., and *Bacteroides* spp. are known to increase PTB (Fettweis et al., 2019). Vaginal dysbiosis, a state of imbalance in the microbial community in the vagina, is related to PTB (Fettweis et al., 2019; Bennett et al., 2020; Kumar et al., 2021). However, the results of microbiome analysis using 16s rRNA metagenome sequencing are difficult to interpret, and since there are many individual differences, it is very difficult to predict PTB using this method.

Many researchers have created prediction models using logistic regression (LR) with PTB-associated clinical information and microbiome data (Hyman et al., 2014; Kumar et al., 2021). Various other machine-learning methods have been applied to classify PTB, such as the random forest (RF) and support vector machine (SVM; Della Rosa et al., 2021; Urushiyama et al., 2021). However, PTB prediction modeling techniques that use intersect markers from several metagenomic analyses have not been studied, and research is lacking on how reproducible the developed models are when applied in practice. Therefore, in this study, we aimed to select markers by analyzing vaginal microbiome data from pregnant women and develop a model with a high predictive rate by combining clinical information.

## Materials and methods

### Study subjects and CVF collection

In this case–control study, subjects were recruited from Yonsei University Severance Hospital and Ewha Womans University Mokdong Hospital between 2018 and 2020. This study was approved by the Ethical Research Committees of Yonsei University Severance Hospital (no. 4-2018-0564) and Ewha Womans University Mokdong Hospital (no. 2018-07-007). All the participants provided written informed consent. The subjects included singleton pregnant women with a gestational age between 17 and 32 weeks. CVF samples were collected from the posterior vaginal fornix using sterile cotton before vaginal examination or clinical treatment, including antibiotics, steroids, and progesterone. For all study subjects, baseline demographic information and health-related characteristics including age, pre-pregnancy body mass index, education level, and maternal PTB history were collected. At the time of CVF sample collection, cervical lengths (CL) were measured, and the white blood cell (WBC) count of the blood test was recorded. After delivery, delivery mode, gestational age at birth (GAB), birth weight of newborn, appearance, pulse, grimace, activity, and respiration (APGAR) scores were evaluated. Subjects diagnosed with gestational diabetes mellitus, preeclampsia, or with insufficient medical records were excluded.

### Metagenome analysis using 16s rRNA gene sequencing

#### Amplification of the V3-4 region of 16S-rRNA gene sequencing for identification of the taxonomy

For microbiome analysis, the collected CVF samples were subjected to bacterial DNA extraction using the NucleoSpin Tissue Kit (Macherey–Nagel, Düren, Germany), following the manufacturer’s instructions. Sequencing of 16S rRNA was performed according to the 16S metagenomic sequencing library preparation protocol, targeting the V3 and V4 hypervariable regions. For PCR and purification of the PCR product, the KAPA HiFi HotStart ReadyMix (KAPA Biosystems, Wilmington, United States) and Agencourt AMPure XP system (Beckman Coulter Genomics, Brea, United States) were used. The initial PCR was performed with 12 ng template DNA using region-specific primers (Supplementary Table 1). After magnetic bead-based purification, a second PCR was performed using primers from the Nextera XT Index Kit (Illumina). Purified PCR products were visualized by gel electrophoresis and quantified using DropSense96 (Trinean, Gentbrugge, Belgium). For quality analysis, the pooled samples were run on an Agilent 2,100 Bioanalyzer (Agilent, Santa Clara, CA, United States). Using the CFX96 Real-Time System, Libraries were quantified by qPCR. After normalization, sequencing of the prepared library was conducted using the MiSeq system (Illumina, San Diego, CA, United States) with 300 bp paired-end reads.

#### Bioinformatics analysis and marker selection

##### Microbiome sequence and composition analysis

Generated paired-end reads were analyzed using DADA2 pipeline (version 1.19.1) to build an amplicon sequence variant (ASV) table (Callahan et al., 2016). Primers which were truncated and the reads with ambiguous bases or more than two expected errors were dropped. The forward and reverse reads were trimmed to 285 and 225, respectively, ensuring a 20 bp overlapping region for the merging step. Taxonomies are assigned to ASV using exact string matching against EzBioCloud 16S database (Yoon et al., 2017). Then, unassigned ASVs were taxonomically identified using NCBI Blast search with 99% sequence similarity. Lastly, ASVs with unidentified taxonomy and low prevalence (<0.005%) were filtered out. Using the Shannon index, the α-diversity was computed to understand the richness and diversity of the microbiome species in the TB and PTB groups (Shannon, 1997). The α-diversity was compared using the Wilcoxon rank-sum test between the two groups. Furthermore, the β-diversity using the Bray-Curtis distance was examined to compare the divergence in the microbiome community between the two groups (Bray and Curtis, 1957).

##### Marker selection

Before developing the prediction models, marker selection was performed to reduce the size of marker set. Samples were split into a training set and a test set in a two-to-one ratio. On the training set, the following seven statistical methods for marker selection were applied: zero-inflated Gaussian mixture model (ZIG), zero-inflated beta regression (ZIBSeq), analysis of microbiome composition (ANCOM), centered log-ratio transformation, and permutation logistic regression model (CLR Permutation), Wilcoxon rank-sum test (Wilcoxon), DESeq2, and edgeR. First, the markers with frequency less than 25% and mean proportion less than 0.001% were filtered out. Then, the markers whose *p-values* were less than 0.05 were selected.

**Figure 1:**
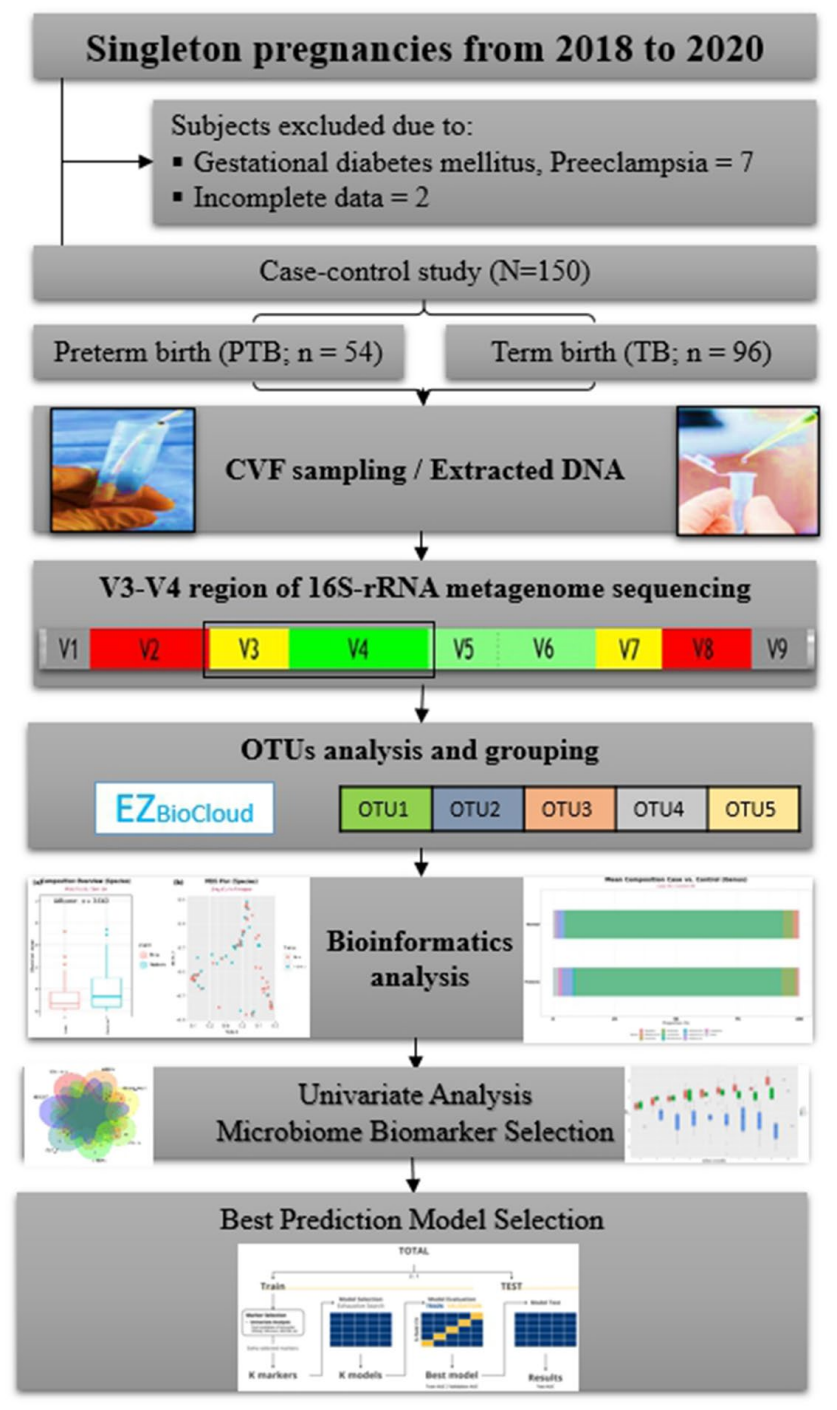
Flowchart of the study. CVF, cervicovaginal fluid; rRNA, ribosomal ribonucleic acid; OTUs, operational taxonomic units.

Among these selected 15 markers, we further investigate whether or not these markers are preterm and genital infection based on the literature searches (Supplementary Table 2). Finally, 10 markers were selected as a marker set. In addition, we also considered the following seven markers that were detected by at least two statistical analyses with its frequencies between 10 and 25%: *Bifidobacterium breve*, *Dialister propionicifaciens*, *Lactobacillus paracasei*, *Mobiluncus curtisii*, *Prevotella disiens*, *S. aureus*, and *Streptococcus anginosus*. Hence, two sets of markers, containing 10 species and 17 species, and the entire marker set were used for the multiple marker selection step (Figure 1).

We performed multiple marker selection in two ways: one from pre-selected sets and the other from the whole marker set. The pre-selected sets were the two sets of statistically significant markers from single marker selection, with or without already reported PTB-related markers. First, for the two pre-selected feature sets, two different feature selection methods were used: exhaustive search and forward selection. We applied five-fold cross-validation (CV) to the training set (Kim et al., 2021). Second, we also performed feature selection from the whole marker set. However, we excluded exhaustive search since the computational cost of exhaustive search increased exponentially on the whole marker set. Instead, we applied stepwise selection and lasso penalization along with forward selection (Tibshirani, 1996; Kim et al., 2019). The detailed multiple marker selection methods are described in Figure 1; Supplementary Figure 1.

##### Prediction model development

LR, RF, XGB, SVM, and GUIDE (version 38.0) were used to develop prediction models (Loh, 2009; Chen and Guestrin, 2016). Hyperparameters of RF, XGB, and SVM were tuned from the training set using five-fold cross-validation. Training set was randomly divided into five separate sets. By using each one as validation set, we trained the model with four other sets and calculated model performance on each of the validation set. The hyperparameters with the greatest mean validation AUCs were chosen. The test set was only used in evaluating the model performances. GUIDE, a single-decision tree-based method, reduced the variable selection bias by choosing significant variables from Chi-square tests (Loh, 2011). The selected split point minimized the node impurity measure. The final tree was pruned using five-fold CV to minimize the misclassification cost. The performance of all the models was measured using the AUC. Then, using the test set, the AUCs of each model were compared to identify a better-performing model.

## Results

### Clinical characteristics

**Figure 2:**
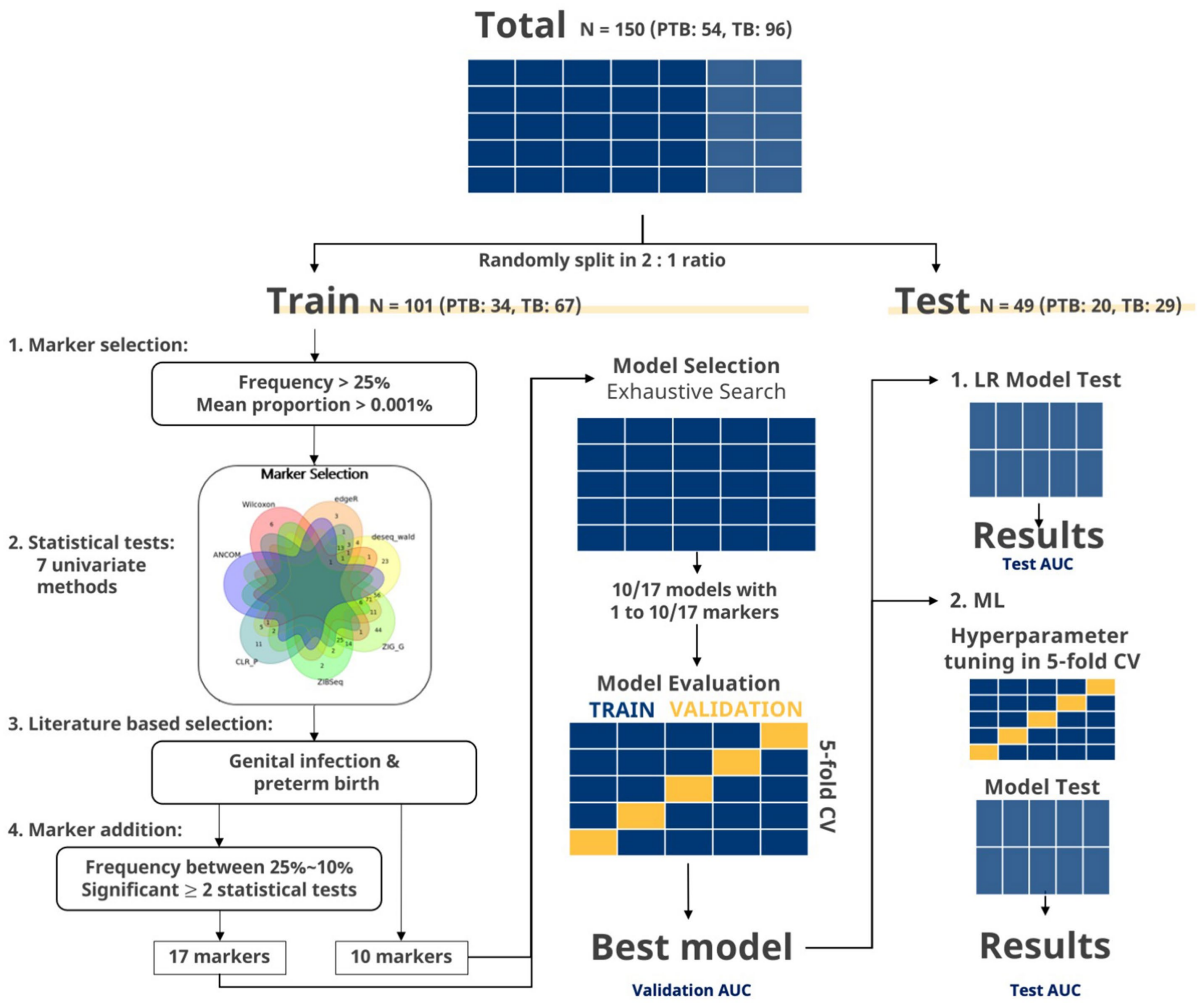
Flowchart of marker selection and evaluation in exhaustive search. The data were split to a training set and test set in a two-to-one ratio. Markers with frequency more than 25% and mean proportion more than 0.001% were selected. Then, markers, showing significant *p* values in two or more statistical tests, were selected. Venn diagram of significant markers (*p* < 0.05) after seven statistical methods (ZIG, ZIBSeq, ANCOM, CLR permutation, Wilcoxon rank-sum test, DESeq2, and edgeR) is shown. Additional filtering steps were applied to the selected markers to finalize the set of 10 and 17 markers. For the given set of markers, exhaustive search was applied to every possible combination of markers using LR. Best marker sets for each number of combinations were selected using AUC from the training set. The global best marker set among these selected sets was chosen as the one that showed the highest AUC from the five-fold CV. Then, the final marker set was select based on the test set. Lastly, the final marker sets were used in building machine learning (ML) models.

A total of 150 women participated in this case–control study: 54 in the PTB group and 96 in the TB group (Figure 2). There were no significant differences between the characteristics of the PTB and TB groups, except for the history of sPTB, WBC count, CL, GAB, birth weight, and APGAR score (Table 1).

**Table 1:** Clinical characteristics of the study subjects.

Characteristics	Preterm birth (*n* = 56)	Term birth (*n* = 99)	*P*-value
Maternal age (year)	32.5 (±3.8)	33.0 (±4.0)	0.427
Pre-pregnancy BMI (kg/m^2^)	21.4 (±3.2)	21.4 (±2.7)	0.938
Education level			>0.999
High school graduation or below	4 (16.0)	11 (15.3)	
University graduates	21 (84.0)	61 (84.7)	
History of PTB			<0.002^*^
No	42 (85.7)	93 (98.9)	
Yes	7 (14.3)	1 (1.1)	
WBC (1 × 10^3^/μl)	11.20 (8.8–13.2)	9.30 (8.0–10.5)	<0.001^*^
GAS (wks)	26.8 (22.8–30.4)	25.8 (22.1–30.5)	0.262
Cervical lengths (mm)	22.7 (13.6–31.9)	30.4 (26.6–36.0)	<0.001^*^
CST type			0.106
I, II, V	18 (36.0)	47 (54.1)	
III	10 (20.0)	19 (21.8)	
IV	22 (44.0)	21 (24.1)	
GAB (wks)	30.6 (27.5–34.1)	38.9 (38.1–39.6)	<0.001^*^
Delivery mode			0.055
ND	25 (44.6)	60 (60.6)	
CS	31 (55.4)	39 (39.4)	
Birth Weight (g)	1738.6 (±885.7)	3234.9 (±323.0)	<0.001*
APGAR score at 1 min	6.23 (3–9)	9.35 (9–10)	<0.001^*^
APGAR score at 5 min	7.55 (6–10)	9.76 (10–10)	<0.001^*^

Categorical variables were expressed as frequencies (percentages) and analyzed using the Chi-square test and Fisher’s exact test. Continuous variables were expressed as mean ± standard deviation (SD) or median (interquartile range) and were compared using the *t*-test or Mann–Whitney *U* test. BMI, body mass index; PTB, preterm birth; WBC, white blood cell; GAS, gestational age at sampling; CST, community-state type; ND, normal delivery; CS, cesarean section; GAB, gestational age at birth; APGAR, appearance, pulse, grimace, activity, respiration; NICU, neonatal intensive care unit.

* p < 0.05, considered statistically significant.

### Association between bacteria and preterm birth

#### Differences in microbial diversity between PTB and TB groups

**Figure 3:**
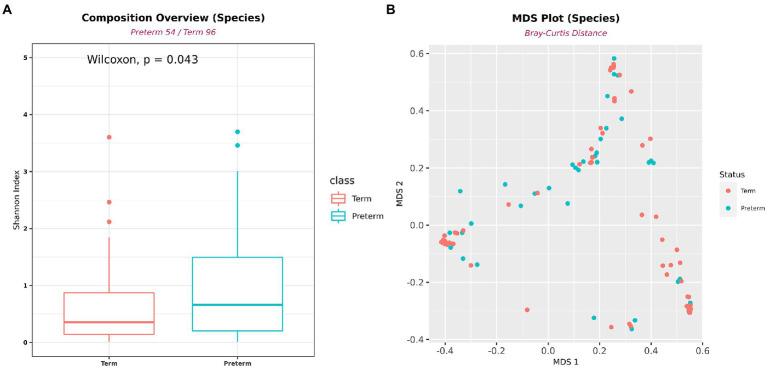
Differences in alpha- and beta-diversity between PTB and TB groups. **(A)** Shannon’s alpha diversity was significantly higher in the PTB group (PTB, *n* = 54; TB, *n* = 96). **(B)** Multidimensional scaling plot. Boxes show median and interquartile ranges, and whiskers extend from minimum to maximum values.

A total of 365 bacteria were detected at the species level. In the diversity analysis of the microbial community, the α-diversity using the Shannon index, an indicator of species diversity, was significantly higher in PTB (Figure 3A). However, there was no significant difference in β-diversity (Figure 3B).

#### Marker selection using univariate analysis

The samples were randomly split into a training set (*n* = 101) and test set (*n* = 49; Supplementary Table 3). Seven different metagenomic analyses were performed on the training set to identify differentially distributed species between the PTB and TB groups. When the marker selection step with mean proportion and frequency was applied, there were 15 markers that showed significance in more than two statistical tests (Figure 1). Additional filtering and literature search were applied to select the following ten species: *Lactobacillus crispatus*, *Lactobacillus fornicalis*, *Lactobacillus gasseri*, *Lactobacillus iners*, *Lactobacillus jensenii*, *G. vaginalis*, *Ureaplasma parvum*, *A. vaginae*, *Prevotella timonensis*, and *Peptoniphilus grossensis* (Supplementary Tables 2, 4). Moreover, seven additional species that could be associated with PTB were appended: *B. breve, D. propionicifaciens, L. paracasei, M. curtisii, P. disiens, S. aureus, and S. anginosus* (Fettweis et al., 2019; Dunlop et al., 2021).

#### Multiple marker selection

LR, exhaustive search, and forward selection were independently applied to the 10 and 17 pre-selected markers to identify the best marker sets. Forward selection, stepwise selection, and LASSO were applied to 365 markers. In addition to these markers, WBC count was included as a covariate. With multiple marker selection, the minimum number of selected markers was one, and the maximum number of selected markers was 49 (Supplementary Table 5). Because the selection result may depend on how the training and test datasets are split, we repeated the entire splitting process 100 times independently. Marker selection was consistent without showing any outlying results (Supplementary Figures 2, 3).

Further analysis was performed on participants with CL information: 67 with TBs and 42 with PTBs. As in the previous analysis, the remaining samples were randomly divided into a training set and a test set with a two-to-one ratio, and multiple marker selections were applied on 10, 17, and the whole marker set. The minimum number of selected variables was 5 and the maximum number of selected variables was 19 (Table 2). We independently repeated the entire splitting process 100 times. Marker selection was consistent without showing any outlying results (Supplementary Figures 4, 5).

**Table 2:** Performances of different multiple marker selection methods and test AUC comparison in prediction models.

		Variables	Train AUC	Validation AUC	Test AUC	LR	RF	XGB	SVM	GUIDE
10 Markers	Best Subset^1^	5	**0.84**	**0.79**	0.68	0.68	**0.74**	**0.77**	**0.74**	**0.73**
	Forward^2^	7	**0.83**	**0.83**	0.66	0.66	0.68	**0.77**	0.66	**0.73**
	Total	12	**0.87**	**0.71**	0.70	0.70	0.63	**0.77**	**0.75**	**0.77**
17 Markers	Best Subset^3^	7	**0.88**	**0.81**	0.57	0.57	0.63	**0.78**	0.59	**0.73**
	Forward^4^	7	**0.83**	**0.83**	0.65	0.65	**0.71**	**0.78**	0.61	**0.73**
	Total	19	**0.95**	0.55	0.60	0.60	0.60	**0.76**	0.60	0.57
365 Markers	Forward^5^	9	**0.98**	**1**	**0.81**	**0.81**	**0.84**	**0.83**	**0.82**	**0.77**
	Stepwise^6^	5	**0.96**	**0.92**	**0.78**	**0.78**	**0.72**	**0.83**	**0.81**	0.63
	Lasso^7^	19	**0.99**	**0.78**	**0.78**	**0.78**	**0.79**	**0.78**	**0.76**	**0.77**

Models with a higher AUC (>0.70) are shown in bold font. Logistic regression (LR), random forest (RF), XGBoost (XBG), support vector machine (SVM), and generalized unbiased interaction detection and estimation (GUIDE) were used to develop the prediction model. CLR-transformed data were used in the LR model and SVM, and proportional data were used for RF and XGB. The markers selected from the different methods are as follows:

1 WBC, cervix length, *Lactobacillus fornicalis, Ureaplasma parvum, Prevotella timonensis*.

2 WBC, cervical length, *U. parvum*, *P. timonensis*, *L. fornicalis*, *Lactobacillus crispatus gallinarum*, *Atopobium vaginae*.

3 WBC, cervical length, *L. crispatus gallinarum, L. fornicalis, U. parvum, Lactobacillus paracasei, and Dialister propionicifaciens*.

4 WBC, cervical length, *U. parvum, P. timonensis, L. fornicalis, D. propionicifaciens, and Mobiluncus curtisii*.

5 WBC, cervical length, *Ureaplasma urealyticum, Alistipes finegoldii, Ruminococcus bromii, PAC001524_s, Peptoniphilus lacrimalis, L. crispatus gallinarum, and Lactobacillus jensenii*.

6 WBC, cervix length, *U. urealyticum, A. finegoldii, R. bromii*.

7 WBC, cervical length, *Prevotella disiens*, *A. finegoldii, Alistipes putredinis*, *PAC001031_s*, *PAC001524_s*, *Peptostreptococcus anaerobius*, *DQ905423_s, PAC001247_s, PAC001402_s, Anaerococcus tetradius, KQ960143_s, P. lacrimalis, Paracoccus marcusii hibiscisoli carotinifaciens, Moraxella osloensis, Pseudomonas glareae benzenivorans, U. parvum, and U. urealyticum*.

#### Prediction model using machine learning algorithms

**Figure 4:**
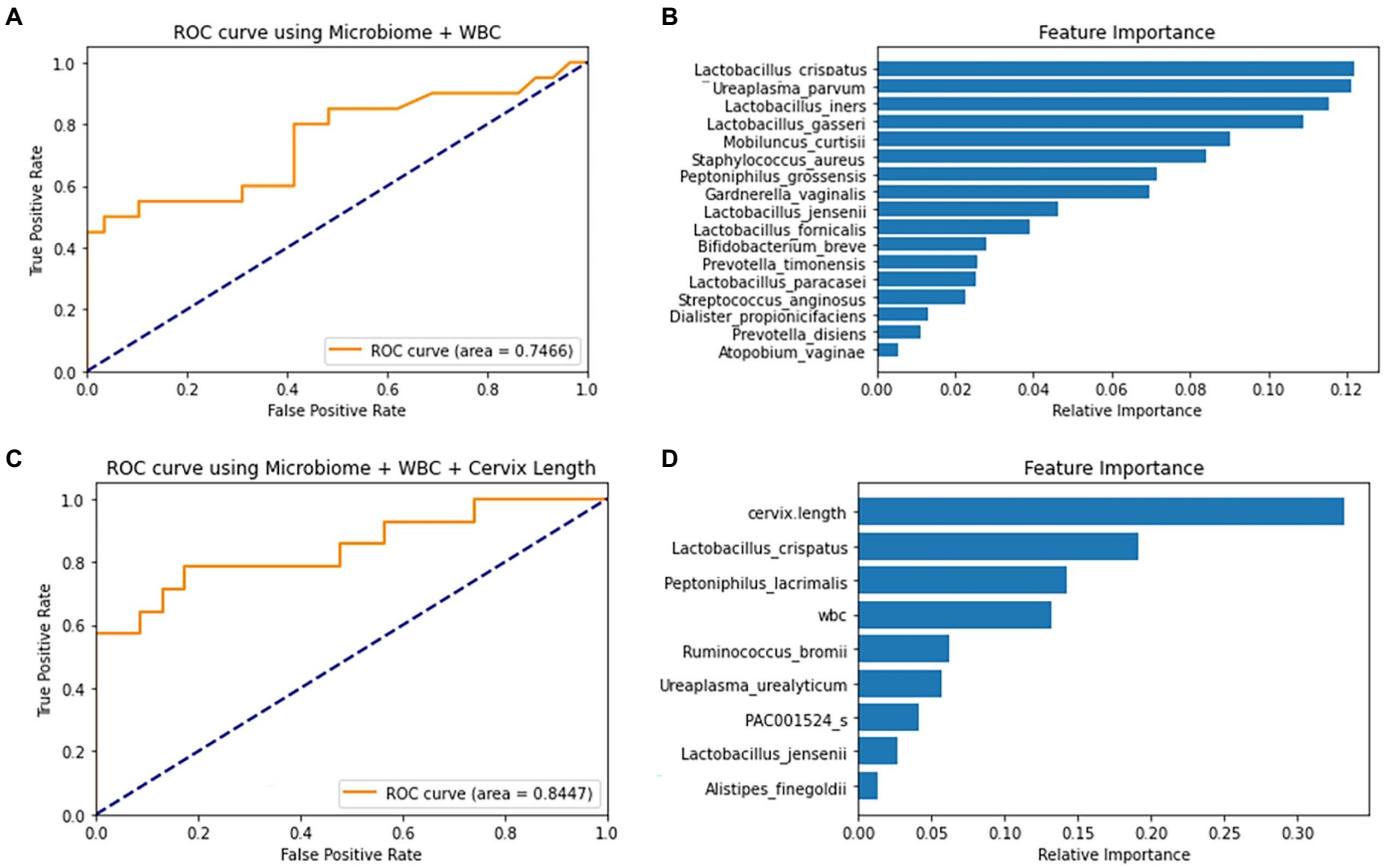
ROC curve and feature importance plot of the Random Forest (RF) models using covariates and selected markers. **(A)** RF model’s ROC curve on test data using 10 selected markers and WBC **(B)** RF model’s feature importance plot using 10 selected markers and WBC. **(C)** RF model’s ROC curve on a test using forward-selected markers, WBC and cervical length. **(D)** RF model’s Feature Importance plot using forward-selected markers, WBC and cervical length.

Using 18 differently selected marker sets, the PTB prediction models were trained based on the following five machine-learning methods. When trained without the covariates, the SVM model using the six markers selected from the 17 pre-selected markers showed the highest test AUC 0.70 (Supplementary Table 5). The RF model using the 17 preselected markers showed a similar AUC of 0.75. *Lactobacillus* spp. and *U. parvum* were reported to be important features for predicting PTB in the RF model (Figures 4A,B; Kataoka et al., 2006; Petricevic et al., 2014; Tabatabaei et al., 2019). When WBC was added as a covariate, most machine-learning methods showed improved prediction performance (Supplementary Table 5).

When PTB prediction models were trained using subjects with CL, those with both WBC and CL generally showed higher test AUCs than those without covariates (Table 2). With the increase of test AUCs, other metrics, such as f1-score and MCC, also increased when the covariates were added to the models (Supplementary Table 6). The RF model using the seven forward selected markers from the total markers showed the highest AUC of 0.84. This model showed a sensitivity of 0.79 when the specificity was 0.83 (Figures 4C,D). In addition, the model’s precision and recall were 0.77 and 0.71, respectively. These precision and recall produced a high f1-score of 0.74, which was the harmonic mean of them. Lastly, the model’s MCC value of 0.59 indicated that there was a positive correlation between model’s prediction and the true value (Supplementary Table 6).

**Figure 5:**
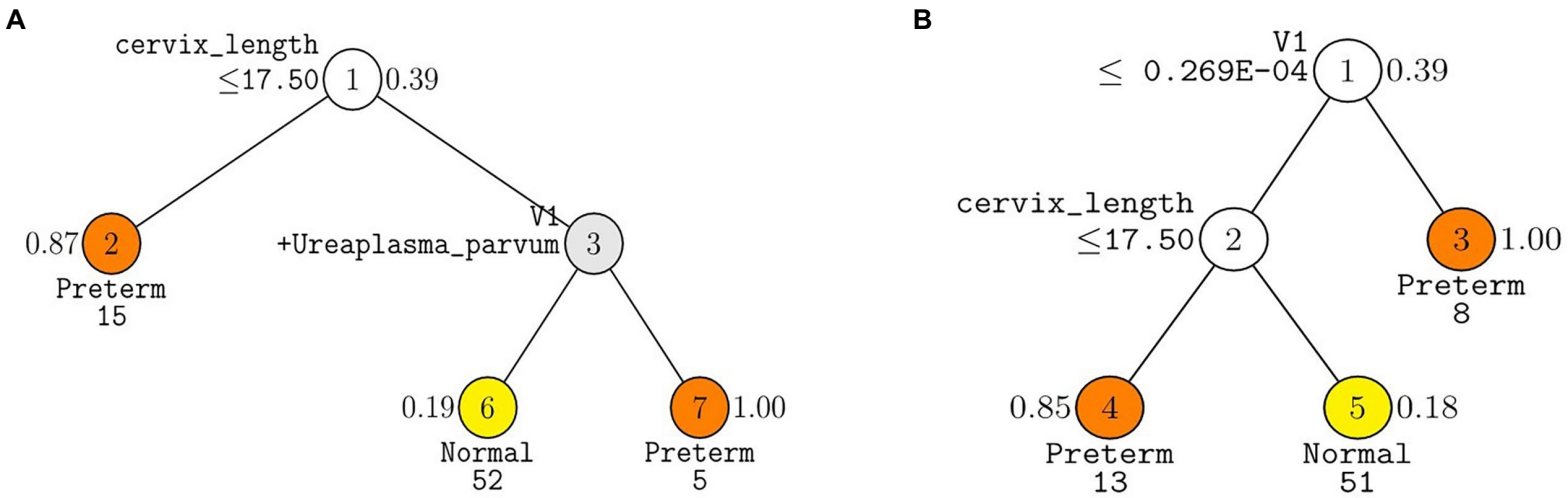
Decision trees made from GUIDE algorithm using covariates, cervix length and WBC with **(A)** ten pre-selected markers and **(B)** seven markers forward selected from the total markers. GUIDE v.38.0 classification tree for predicting Y using estimated priors and unit misclassification costs. Tree constructed with 109 observations. Pruning parameter *α* was 0.02 for **A** and 0.03 for **B**. At each split, an observation goes to the left branch if and only if the condition is satisfied. Predicted classes and sample sizes printed below terminal nodes; class sample proportion for Y = Preterm beside nodes. In **(A)**, V1 stands for *Prevotella timonensis*. In **(B)** V1 stands for *Peptoniphilus lacrimalis.*

The following three GUIDE models yielded the highest test AUC of 0.77 using (1) 10 pre-selected markers, (2) 7 markers forward selected from the total markers, and (3) 17 markers selected from the total markers *via* LASSO (Table 2). In the GUIDE method, when 10 selected markers and CL were used, cases with a CL < 17.5 mm were highly related to PTB, and in cases of CL > 17.5 mm, when *Ureaplasma* and *Prevotella* increased, there was a tendency toward PTB (Figure 5A). Among the markers selected by forward selection and LASSO, an increase in *Peptoniphilus lacrimalis* showed a high association with PTB when CL <17.5 mm (Figure 5B).

## Discussion

This is the first study using a machine learning technique to predict PTB using vaginal microbiome, blood WBC, and CL, suggesting that the generated prediction model could be used to predict PTB considering model validation. In this study, the vaginal microbiome was analyzed using 16s rRNA metagenome sequencing.

In most microbiome studies with 16s rRNA metagenome sequencing, an operational taxonomic unit (OTU) is commonly used. OTU is derived from a cluster of similar sequences, while ASV is inferred from a unique sequence. Hence, ASVs can define sequences in one nucleotide difference and provide finer resolution (Callahan et al., 2017). In addition, their representations of sequences do not depend on the choice of reference database, because the inference is not computed based on the reference database but on the sequences (Callahan et al., 2017). AST inference is computed by separating technical errors and biological differences. Learning error rates and denoising errors are essential in making an ASV table. We chose to use DADA2 pipeline, because it is most popularly used to estimate error rates and statistically denoise the errors in the sequences (Callahan et al., 2016).

Candidate markers, used in training machine learning models, were selected through a combination of various methods, such as literature search and numerous statistical tests. As we focused on building machine learning models that can accurately predict preterm births, we chose a wider range of markers for the models by using a less stringent threshold in the statistical tests. To avoid any possible positive errors, we selected differentially abundant markers commonly detected by at least two statistical methods. Then, 17 markers were selected using a multiple marker selection method that can predict PTB (AUC = 0.78). Markers selected using forward selection, stepwise selection, and LASSO showed significant performance (AUC = 0.84).

We chose use AUC because it has advantages of comparing models with a combination of sensitivity and specificity in all decision thresholds. As AUC represents area under a curve that is drawn from all possible combinations of sensitivity and specificity, models with high AUC will have high sensitivity and specificity.

In model comparison, the models using various marker selection methods from the total markers set resulted in best predictions across a large portion of the model (Table 2). It is because these models utilized not only the statistically significant markers from 10 and 17 markers sets but also additional markers not selected in the statistical tests. These additional markers were chosen through forward/stepwise selection and lasso as they enhanced model performances. For example, *Moraxella osloensis* and *P. lacrimalis* were filtered out according to our filtering criteria but they were selected in the marker selection from the total markers set. As a result, the models using the total marker set resulted in the best predictions across a large portion of the models.

This study validated the prediction model by evaluating its performance on the test set. Using the test set, it was possible to predict the model performance for future patients. Furthermore, using the GUIDE method, it was possible to determine the role of each microbiome. Through the presentation of the tree using the GUIDE method, it was confirmed that the association with PTB was high when *Prevotella* and *Ureaplasma* increased, and it could also be explained that, as the number of *P. lacrimalis* increased, the association with PTB was higher (Figure 4).

The findings of this study showed similarity to those of various vaginal microbiome studies that used 16s rRNA metagenome sequencing. There have been several reports showing that *Lactobacillus* is related to PTB, and it is known that the risk of PTB is high in the group in which *Lactobacillus* is not dominant (Fettweis et al., 2019). If the bacterial diversity is high, the distribution of other pathogens increases, resulting in an increased risk of PTB (Fox and Eichelberger, 2015; Chu et al., 2018; Dominguez-Bello, 2019; Oliver et al., 2020). Various bacteria related to PTB have been reported (Freitas et al., 2018; Romero et al., 2019; You et al., 2019; Payne et al., 2020; Sprong et al., 2020); however, this study suggested that *Lactobacillus* spp. *U. parvum, M. curtisii, S. aureus,* and *Peptoniphilus grossensis* played a more important role, and the GUIDE method explained that *P. timonensis, U. parvum,* and *P. lacrimalis* played the most important role in PTB. The measurement of CL was performed to predict the risk of PTB in the second trimester globally. If it is shorter than 25 mm, it is considered high-risk, and if it is less than 15 mm, it is recommended to be hospitalized (Suff et al., 2019). The standard suggested by GUIDE in this study was 17.5 mm, and similarly, if it was shorter than the standard, the risk of PTB was high.

In this study, as a noninvasive method, the possibility of developing a PTB prediction model using the microbiome analysis results of CVF, blood test results, and CL measurement was shown. In addition, our study showed better predictive power than the existing method of PTB prediction. Fetal fibronectin (fFN) is commonly used in clinical applications for PTB prediction (Heng et al., 2015). However, the sensitivity of fFN is only 0.56 and that of phosphorylated insulin-like growth factor binding protein-1 (phIGFBP1) is 0.33 (Conde-Agudelo et al., 2011). In comparison, the PTB prediction model developed in this study showed a better sensitivity of 0.79 and specificity of 0.83. As a result, the application of this prediction model, based on the most important microbiome, could be clinically useful and cost-effective.

In this study, we compared the best prediction methods using various marker selection techniques and machine-learning methods. Although RF and XGB provide better prediction performance, they lack reasonable biological interpretation. For instance, in XGB model with forward selected markers from entire marker set, it is possible to observe each feature’s impact by applying SHAP method (Supplementary Figure 6). However, it is difficult to interpret the model in relation to whole features. On the other hand, a simple single-tree model is easier to interpret, but is also known to have lower performance than other tree ensemble models (Hasan et al., 2020). The GUIDE, an enhanced version of a single tree, can still be used for the sake of interpretability with improved performance (Figure 4).

In future research, prediction models can be applied in clinical practice through a method that can more quantitatively evaluate the microbiome relationship, or it may be useful to substitute PCR tests that can utilize whether mRNA is expressed from DNA (Loh, 2014; Payne et al., 2020). To confirm the biological mechanism, it may be necessary to study proteomics and metabolomics in addition to genomics. Furthermore, studies on changes in cytokines or immune activation to determine how this microbiome acts with the host should be conducted.

Previous studies that presented the predictive power of machine learning models neglected to present the test AUC (Pedregosa et al., 2011; Hyman et al., 2014; Kumar et al., 2021; Urushiyama et al., 2021). This study confirmed the predictive power of the models on the test set. Therefore, this study presents a more accurate predictive power.

As the causes of PTB are very diverse, this was an attempt to increase predictive power by taking a combined approach that looked at the patient’s blood test results and changes in CL, rather than a simple microbiome analysis (Park et al., 2021). In the marker selection process, we implemented an evidence-based medicine method through the existing literature review to select markers related to preterm birth, which can properly complement the machine-learning method using the data-driven hypothesis (Lamont et al., 2020). This study has strengths as it was a large-scale, multicenter study targeting pregnant Korean women. In addition, because the microbiome differs between races, it was possible to identify species related to PTB in Korea.

However, the limitations of this study are that the entire microbiota was not analyzed, including strain level measurements for *U. parvum*, despite recent studies showing that the pathogenicity of *Ureaplasma* differs depending on serovar levels. As a limitation of the method itself, 16s rRNA metagenome sequencing can analyze all colonized microbiomes of the vagina with high sensitivity, but it is difficult to identify the actual activity and pathogenicity of the microbiome. In addition to measuring CL to predict preterm birth, recently, a method of predicting preterm birth using elastography has been widely used (Seol et al., 2020), but this method was not applied in this study.

Our study demonstrates that several candidate microbiota could be used as potential predictors for PTB, and we confirmed that the predictive rate can be increased through a machine learning model based on the cervical length and WBC count.

## Data availability statement

The data presented in the study are deposited in the SRA repository, accession number PRJNA845012.

## Ethics statement

The studies involving human participants were reviewed and approved by this study was approved by the Ethical Research Committee of Ewha Womans University Mokdong Hospital (no. 2018-07-007) and Yonsei University Severance Hospital (no. 4-2018-0564), and all participants provided written informed consent. The patients/participants provided their written informed consent to participate in this study.

## Author contributions

SP enrolled subjects and wrote and edited the manuscript. JM wrote the manuscript and analyzed data. NK analyzed data and interpreted data. Y-HK enrolled subjects and designed the study. Y-AY developed the extraction of protocols and interpreted analyzed data. EK and AA developed the extraction of protocols and performed the experiments. YH enrolled subjects. YK designed the study, obtained funding, and enrolled subjects. TP designed the study and obtained funding. All authors contributed to the article and approved the submitted version.

## Funding

This study was supported by the National Research Foundation of Korea (NRF) grant funded by the Korean government (MSIT; grant no. 2020R1A2C3011850) and the BK21 FOUR (Fostering Outstanding Universities for Research) funded by the Ministry of Education (MOE, Korea) and National Research Foundation of Korea (NRF). This work was also supported by a grant from the Korea Health Technology R&D Project through the Korea Health Industry Development Institute (KHIDI), funded by the Ministry of Health & Welfare, Republic of Korea (grant numbers: HI16C2037).

## Conflict of interest

The authors declare that the research was conducted in the absence of any commercial or financial relationships that could be construed as a potential conflict of interest.

## Publisher’s note

All claims expressed in this article are solely those of the authors and do not necessarily represent those of their affiliated organizations, or those of the publisher, the editors and the reviewers. Any product that may be evaluated in this article, or claim that may be made by its manufacturer, is not guaranteed or endorsed by the publisher.
