# Supplementary material for: Predicting preterm birth through vaginal microbiota, cervical length, and WBC using a machine learning model
Source: Front Microbiol. 2022 Aug 2;13:912853. doi: 10.3389/fmicb.2022.912853 (PMC9378785; doi:10.3389/fmicb.2022.912853)
Supplement: Supplementary file 1 [file Data_Sheet_1.docx]

**Supplementary Table 1.** Region-specific primers

| Forward primer | 5′-TCGTCGGCAGCGTCAGATGTGTATAAGAGACAGTCGTCGGCAGCGTCAGATGT  GTATAAGAGACAGCCTACGGGNGGCWGCAG-3′ |
| --- | --- |
| Reverse primer | 5′-GTCTCGTGGGCTCGGAGATGTGTATAAGAGACAGGTCTCGTGGGCTCGGAGA  TGTGTATAAGAGACAGGACTACHVGGGTATCTAATCC-3′ |

**Supplementary Table 2.** Description of literature-sourced markers. Sequencing technologies, bioinformatic pipelines, and taxonomy databases of the literatures are described accordingly. Countries where the research had been done are also described in abbreviation.

| Species | Sequencing | Database | Country | Source |
| --- | --- | --- | --- | --- |
| *Atopobium vaginae* | qPCR  16s rRNA  16s rRNA | -  RDP  RDP | FR  US  JP | Menard et al., 2010  Ma and Li, 2017  Urushiyama et al., 2021 |
| *Gardnerella vaginalis* | qPCR  16s rRNA  16s rRNA  16s rRNA  qPCR  16s rRNA | -  cpn60  RDP  Silva  -  Silva | FR  CA  ES  US  AU  TH | Menard et al., 2010  Freitas et al., 2018  Cobo et al., 2019  Romero et al., 2019  Payne et al., 2020  Kumar et al., 2021 |
| *Lactobacillus spp.* | 16s rRNA  Gell  Review  16s rRNA  16s rRNA  16s rRNA  16s rRNA | -  -  -  RDP  PECAN  Greengenes  StrainInfo | US  AT  -  ES  US  US  KR | Ghartey et al., 2014  Petricevic et al., 2014  Aldunate et al., 2015  Cobo et al., 2019  Elovitz et al., 2019  Fettweis et al., 2019  You et al., 2019 |
| *Peptoniphilus grossensis* | 16s rRNA  16s rRNA  16s rRNA | RDP  Silva  RDP | ES  US  JP | Cobo et al., 2019  Romero et al., 2019  Urushiyama et al., 2021 |
| *Prevotella spp.* | 16s rRNA  16s rRNA  16s rRNA | cpn60  Greengenes  Silva | CA  US  TH | Freitas et al., 2018  Fettweis et al., 2019  Kumar et al., 2021 |
| *Ureaplasma parvum* | Cell culture  Cell culture  16s rRNA  16s rRNA  qPCR | -  -  RDP  Silva  - | GE  BE  ES  US  AU | Abele-Horn et al., 2000  Breugelmans et al., 2010  Cobo et al., 2019  Romero et al., 2019  Payne et al., 2020 |
| *Bifidobacterium breve* | 16s rRNA  16s rRNA | RDP  GAST | JP  CA | Matsumoto et al., 2018  Tabatabaei et al., 2019 |
| *Dialister propionicifaciens* | 16s rRNA  16s rRNA | PECAN  RDP | US  JP | Dunlop et al., 2021  Urushiyama et al., 2021 |
| *Mobiluncus curtisii* | 16s rRNA  Cell culture | PECAN  - | US  - | Elovitz et al., 2019  Dude et al., 2020 |
| *Staphylococcus aureus* | 16s rRNA  - | GenBank  - | GE  UA | Kikhney et al., 2017  Salmanov et al., 2021 |
| *Streptococcus anginosus* | 16s rRNA  16s rRNA  16s rRNA | RDP  Silva  RDP | ES  US  JP | Cobo et al., 2019  Romero et al., 2019  Urushiyama et al., 2021 |

**Supplementary Table 3.** Splits of subjects in the training set and the test set (Pearson's Chi-squared p-value: 0.5).

|  | **Train** | **Test** | **Total** |
| --- | --- | --- | --- |
| **Normal** | 67 | 29 | 96 |
| **Preterm** | 34 | 20 | 54 |
| **Total** | 101 | 49 | 150 |

**Supplementary Table 4.** Univariate test results for the PTB and TB groups in the training set. The top 10 markers were 10 selected marker sets, and the bottom 7 markers were additional markers included in the 17 selected marker sets

| Species | Wilcoxon | edgeR | DESeq2_Wald | ZIG_Gaussian | ZIBSeq | CLR_Perm | ANCOM | Sig. | Mean prop. Preterm | Mean prop. Term | Freq. Preterm | Freq. Term |
| --- | --- | --- | --- | --- | --- | --- | --- | --- | --- | --- | --- | --- |
| *Atopobium vaginae* | 0.7842 | **0.0186** | 0.8344 | 0.6100 | 0.7457 | 0.7970 | - | 1 | 1.41% | 2.65% | 32.35% | 31.34% |
| *Gardnerella vaginalis* | 0.1993 | 0.7422 | 0.5523 | 0.2330 | 0.7421 | 0.1450 | - | 0 | 9.20% | 7.25% | 64.71% | 47.76% |
| *Lactobacillus crispatus/gallinarum* | 0.4677 | **0.0202** | 0.6629 | 0.5154 | 0.6323 | 0.5470 | - | 1 | 37.55% | 43.51% | 100.00% | 98.51% |
| *Lactobacillus fornicalis* | 0.8534 | 0.9639 | 0.5151 | 0.1590 | 0.6166 | 0.8180 | - | 0 | 2.57% | 3.66% | 50.00% | 38.81% |
| *Lactobacillus gasseri* | 0.1697 | **0.0000** | **0.0025** | **0.0375** | 0.7857 | 0.0630 | - | 3 | 3.43% | 1.47% | 50.00% | 28.36% |
| *Lactobacillus iners* | 0.9618 | 0.8378 | 0.3708 | 0.5019 | 0.5352 | 0.9860 | - | 0 | 19.13% | 25.46% | 85.29% | 77.61% |
| *Lactobacillus jensenii* | 0.7895 | 0.0718 | **0.0003** | 0.8830 | 0.7157 | 0.7130 | - | 1 | 0.32% | 1.80% | 26.47% | 25.37% |
| *Peptoniphilus grossensis* | 0.4307 | **0.0016** | **0.0000** | **0.0001** | 0.2771 | **0.0270** | - | 4 | 0.70% | 0.01% | 26.47% | 14.93% |
| *Prevotella timonensis* | 0.5698 | 0.0823 | **0.0002** | 0.0983 | 0.9696 | 0.3600 | - | 1 | 0.16% | 0.03% | 32.35% | 23.88% |
| *Ureaplasma parvum* | 0.5838 | 0.7406 | **0.0004** | **0.0144** | 0.1356 | 0.2150 | - | 2 | 1.03% | 0.17% | 44.12% | 37.31% |
| *Bifidobacterium breve* | 0.8534 | **0.0002** | **0.0011** | 0.3444 | 0.6101 | 0.7620 | - | 2 | 0.23% | 0.55% | 17.65% | 11.94% |
| *Dialister propionicifaciens* | 0.8695 | 0.4435 | 0.2360 | **0.0065** | 0.8575 | 0.1850 | - | 1 | 0.03% | 0.02% | 20.59% | 7.46% |
| *Lactobacillus paracasei* | 0.9509 | **0.0005** | **0.0000** | **0.0001** | 0.6997 | 0.8330 | - | 3 | 0.10% | 0.23% | 20.59% | 16.42% |
| *Mobiluncus curtisii* | 0.2219 | **0.0019** | **0.0000** | **0.0000** | 0.2237 | **0.0060** | - | 4 | 0.02% | 0.00% | 23.53% | 5.97% |
| *Prevotella disiens* | 0.7842 | **0.0348** | **0.0000** | **0.0000** | 0.5272 | 0.0890 | - | 3 | 0.17% | 0.00% | 14.71% | 7.46% |
| *Staphylococcus aureus* | 0.0692 | 0.5542 | 0.7621 | **0.0439** | 0.4373 | 0.2540 | - | 1 | 0.08% | 0.07% | 23.53% | 35.82% |
| *Streptococcus anginosus* | 0.9400 | **0.0007** | **0.0000** | **0.0000** | 0.4052 | 0.2190 | - | 3 | 0.18% | 0.00% | 14.71% | 11.94% |

**Supplementary Table 5.** Performance of different multiple marker selection methods using LR for all samples (N=150). Models with WBC generally showed a higher AUC in both the five-fold cross-validation and the test set. Test AUC comparison in prediction models; logistic regression (LR), random forest (RF), XGBoost (XBG), support vector machine (SVM), and GUIDE. CLR-transformed data were used in the LR model and SVM, and relative abundance data were used in RF, XGB, and GUIDE

|  |  |  | var | | trAUC | | valAUC | teAUC | LR | | RF | | XGB | SVM | | GUIDE |
| --- | --- | --- | --- | --- | --- | --- | --- | --- | --- | --- | --- | --- | --- | --- | --- | --- |
| - WBC | **10 Markers** | Best Subset | 3 | 0.66 | | 0.61 | | 0.53 | 0.53 | 0.65 | | 0.66 | | | 0.41 | 0.53 |
|  |  | Forward | 3 | 0.63 | | 0.64 | | 0.62 | 0.62 | 0.67 | | 0.49 | | | 0.54 | 0.5 |
|  |  | Total | 10 | **0.73** | | 0.54 | | 0.54 | 0.54 | 0.67 | | 0.57 | | | 0.61 | 0.5 |
|  | **17 Markers** | Best Subset | 6 | **0.74** | | 0.67 | | 0.47 | 0.47 | 0.59 | | 0.62 | | | **0.70** | 0.53 |
|  |  | Forward | 1 | 0.61 | | 0.64 | | **0.72** | **0.72** | 0.65 | | 0.50 | | | 0.42 | 0.5 |
|  |  | Total | 17 | **0.77** | | 0.54 | | 0.5 | 0.5 | 0.69 | | 0.62 | | | 0.45 | 0.53 |
|  | **365 Markers** | Forward | 21 | **0.95** | | **0.96** | | 0.68 | 0.68 | 0.66 | | 0.64 | | | 0.68 | 0.66 |
|  |  | Stepwise | 7 | **0.92** | | **0.88** | | 0.62 | 0.62 | 0.63 | | 0.59 | | | 0.62 | 0.47 |
|  |  | Lasso | 4 | **0.76** | | 0.67 | | 0.55 | 0.55 | 0.60 | | 0.52 | | | 0.57 | 0.55 |
| + WBC | **10 Markers** | Best Subset | 4 | **0.74** | | **0.71** | | 0.67 | 0.67 | 0.61 | | 0.56 | | | 0.51 | 0.56 |
|  |  | Forward | 4 | **0.74** | | **0.71** | | **0.73** | **0.73** | 0.62 | | 0.64 | | | 0.60 | 0.56 |
|  |  | Total | 11 | **0.78** | | 0.63 | | 0.66 | 0.66 | **0.72** | | 0.62 | | | **0.73** | 0.56 |
|  | **17 Markers** | Best Subset | 4 | **0.74** | | **0.71** | | 0.67 | 0.67 | 0.61 | | 0.66 | | | 0.51 | 0.56 |
|  |  | Forward | 5 | **0.74** | | **0.71** | | 0.67 | 0.67 | 0.58 | | 0.62 | | | 0.62 | 0.56 |
|  |  | Total | 18 | **0.81** | | 0.60 | | 0.63 | 0.63 | **0.75** | | 0.64 | | | **0.70** | 0.58 |
|  | **365 Markers** | Forward | 16 | **0.96** | | **0.96** | | 0.68 | 0.68 | 0.68 | | 0.58 | | | 0.70 | 0.55 |
|  |  | Stepwise | 10 | **0.95** | | **0.87** | | 0.67 | 0.67 | 0.53 | | 0.62 | | | 0.69 | 0.55 |
|  |  | Lasso | 49 | **1** | | 0.67 | | **0.71** | **0.71** | 0.66 | | 0.66 | | | **0.74** | 0.61 |

**Supplementary Table 6.** Model performance comparison in various informative metrics. Models were trained and tested using training and test set from 109 samples, respectively. Metrics used to describe model performance are AUC, accuracy, balanced accuracy, precision, recall, f1-score, and Matthew’s correlation coefficient (MCC). These metrics generally increased when the covariates were added to the models.

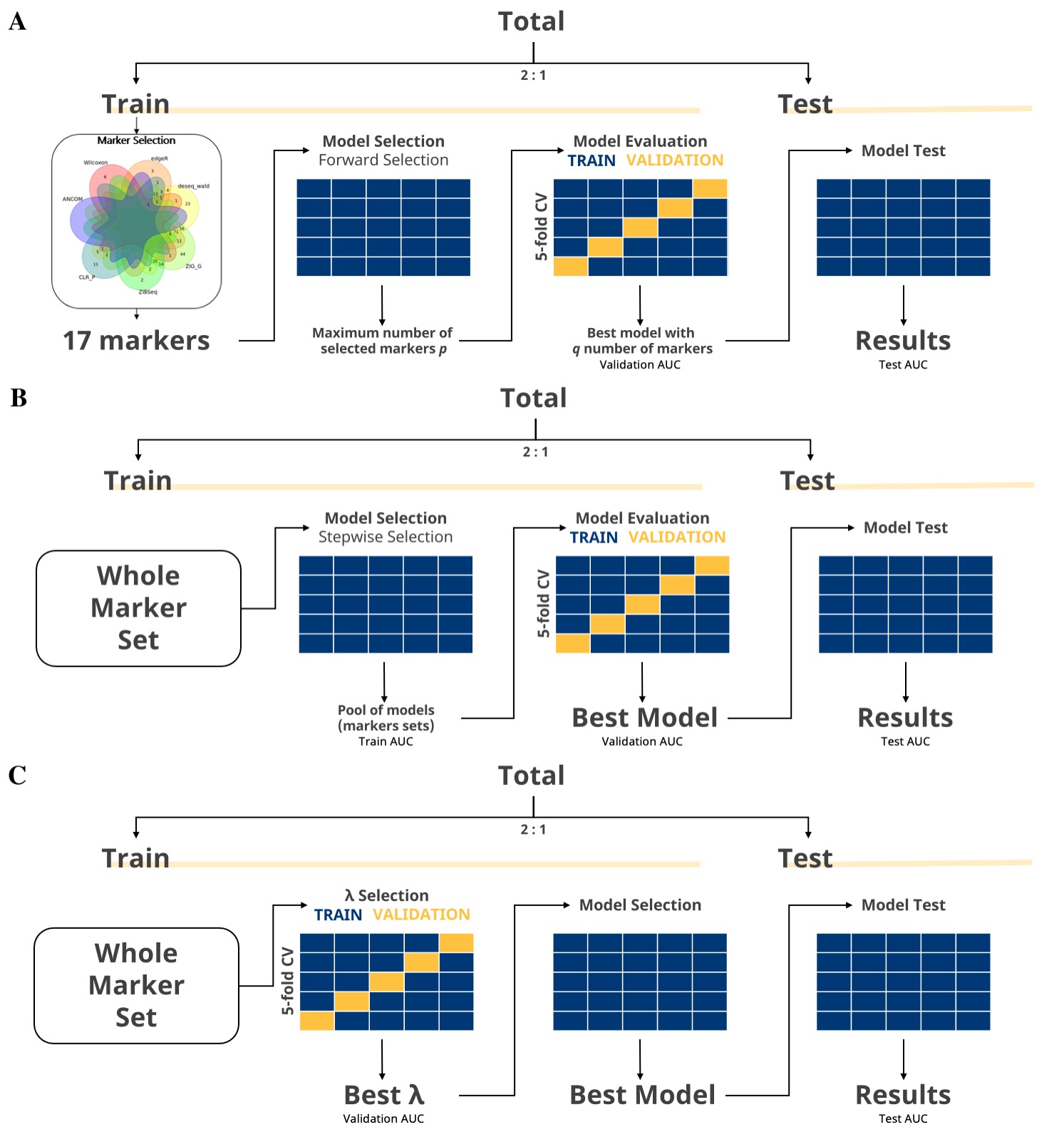


**Supplementary Figure 1.** Flow chart of multiple marker selection and evaluation in forward selection, stepwise selection, and lasso. The data was split to a training set and a test set into two to one ratio. (**A**) The forward selection method was applied to the training set to fix a maximum number of selected marker *p*. Then, the selection procedure was continued until the five-fold CV AUC did not increase. Then, the number of markers *q* is chosen from the model that showed the highest mean validation AUC. Lastly, the combination with *q* number of markers selected in the training data are chosen as the final marker set. (**B**) We applied the stepwise selection procedure to the training set to to obtain a pool of marker sets. Among the pool of marker sets from each step, the best marker set was chosen as the one that showed the highest AUC from the five-fold CV. (**C**) We applied lasso method to the training set. The tuning parameter λ with the highest AUC was chosen from the five-fold CV. With the selected λ, the final model was fitted using the whole training data. The selected variables in the final model were chosen as the final marker set.

**
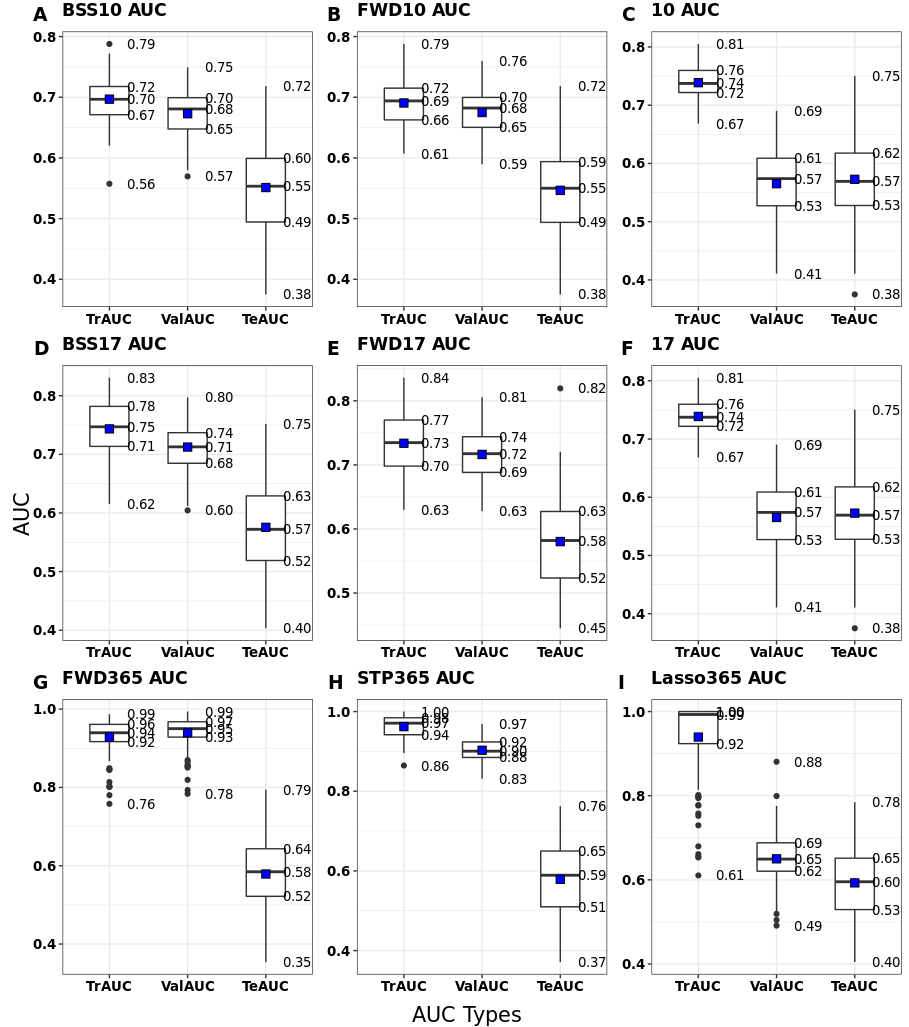
Supplementary Figure 2.** Box plots of 100 AUC on different multiple marker selection methods without using covariate. 150 samples were divided into train set and test set with a two to one ratio. This step was repeated 100 times to generate different splits.

**
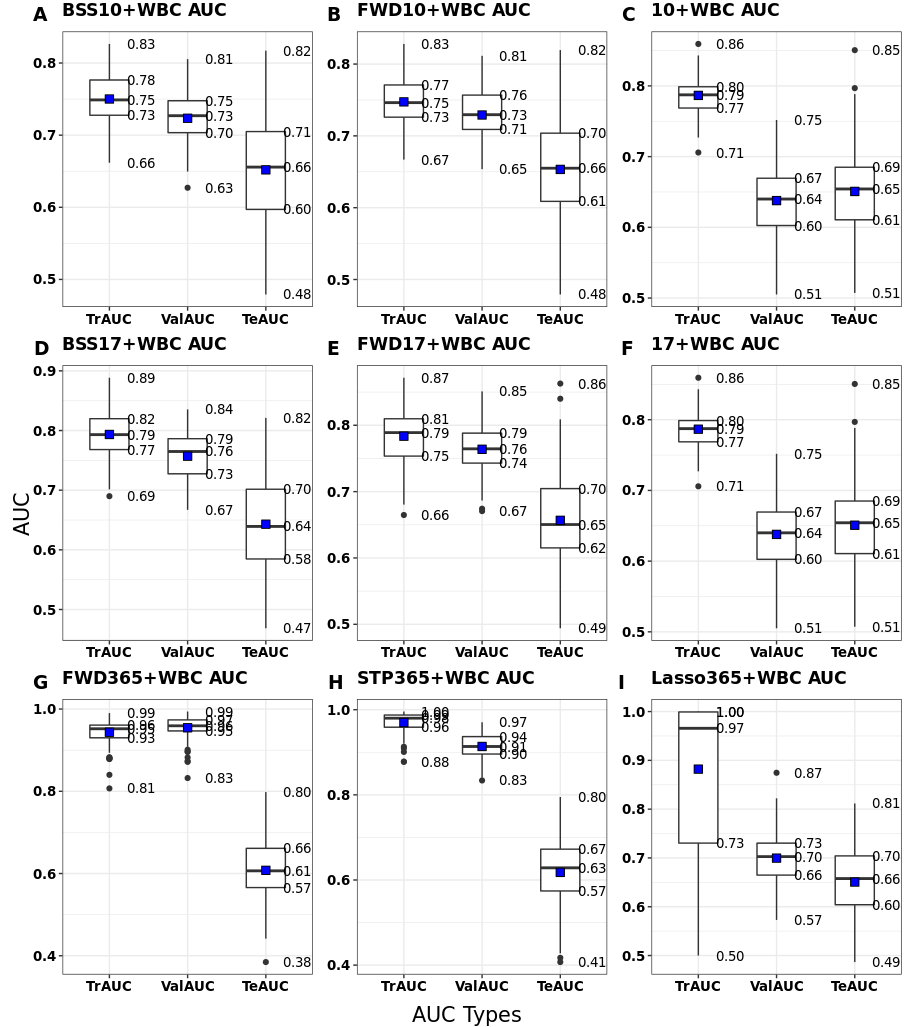
Supplementary Figure 3.** Box plots of 100 AUC on different multiple marker selection methods using covariate. 150 samples were divided into train set and test set with a two to one ratio. This step was repeated 100 times to generate different datasets.

**
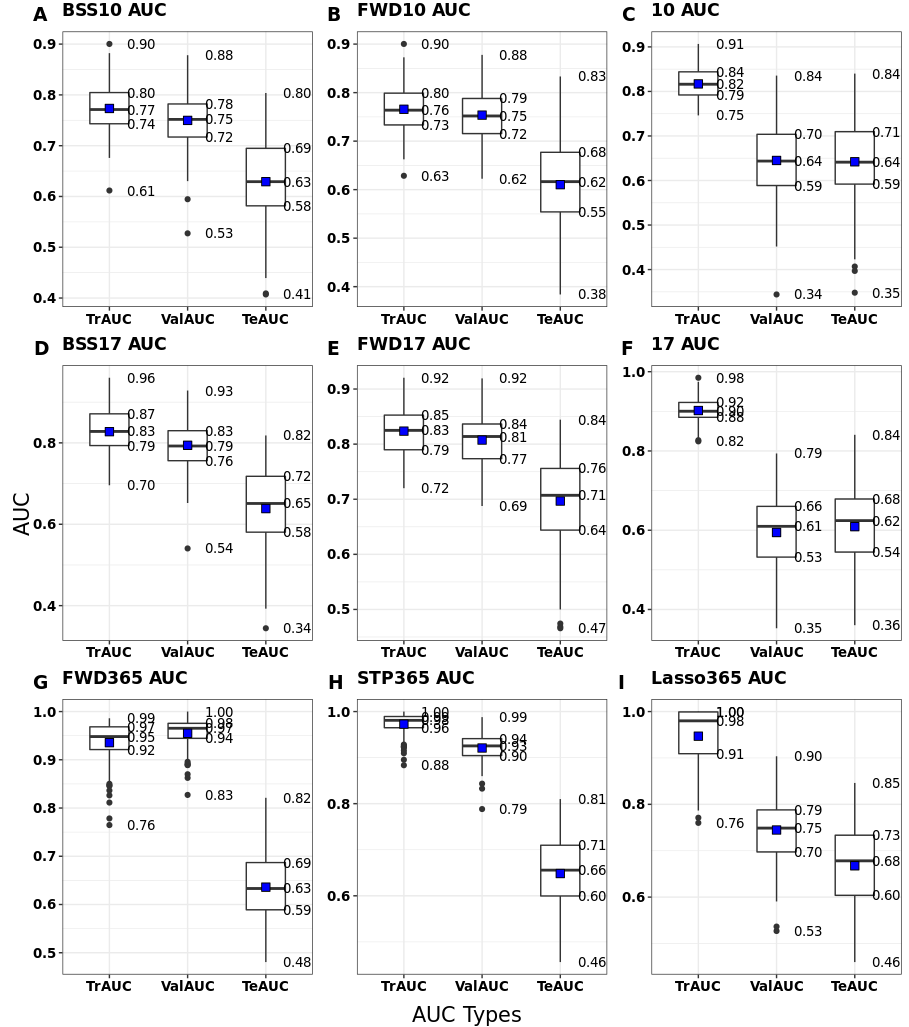
Supplementary Figure 4.** Box plots of 100 AUC on different multiple marker selection methods without using covariate. 109 samples were divided into train set and test set with a two to one ratio. This step was repeated 100 times to generate different datasets.


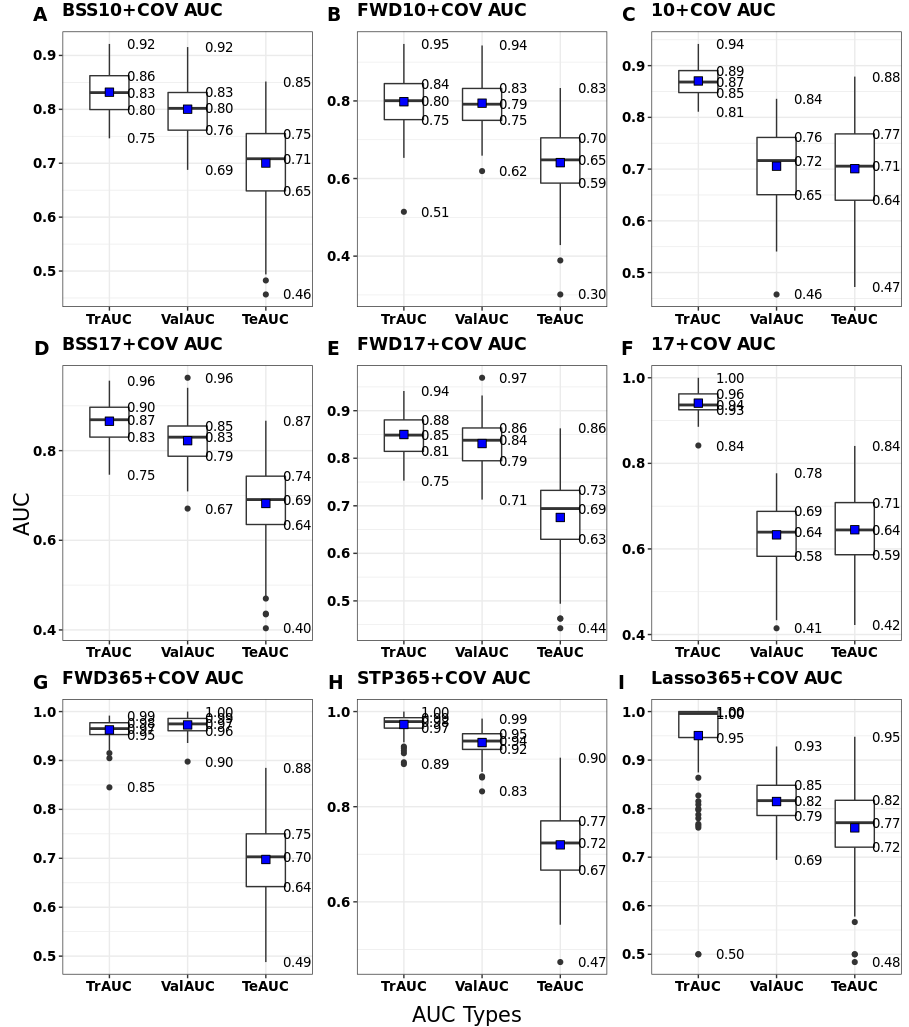


**Supplementary Figure 5.** Box plots of AUC on different multiple marker selection methods using covariate. 109 samples were divided into train set and test set with a two to one ratio. This step was repeated 100 times to generate different datasets.


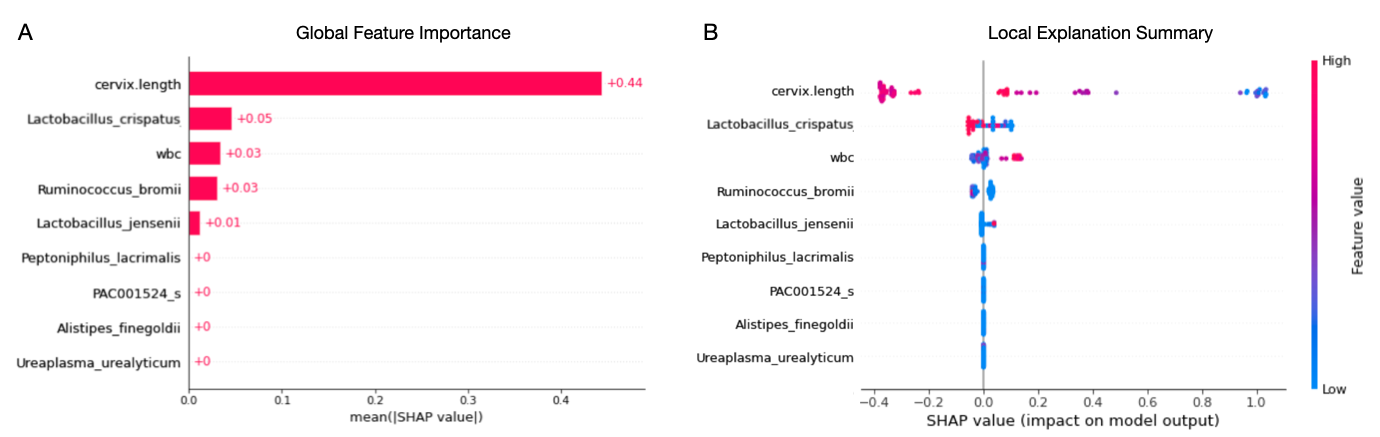


**Supplementary Figure 6.** SHAP interpretation of XGB model with forward selected markers from entire marker set. Global feature importance plot (A) represents mean absolute SHAP values for each feature. Local explanation summary (B) shows feature importance with its effect. The x-axis represents SHAP values and colors represent feature values. In the figure A, cervix length, followed by *Lactobacillus crispatus*, has the most contribution to the prediction. In figure B, shorter cervix length contributes to preterm birth more than the longer ones and having low proportion of *Lactobacillus crispatus* increases risk of preterm birth.
